# Object-Oriented Classification of Sugarcane Using Time-Series Middle-Resolution Remote Sensing Data Based on AdaBoost

**DOI:** 10.1371/journal.pone.0142069

**Published:** 2015-11-03

**Authors:** Zhen Zhou, Jingfeng Huang, Jing Wang, Kangyu Zhang, Zhaomin Kuang, Shiquan Zhong, Xiaodong Song

**Affiliations:** 1 Institute of Remote Sensing and Information Application, Zhejiang University, Hangzhou, China; 2 Key Laboratory of Agricultural Remote Sensing and Information System, Zhejiang Province, Hangzhou, China; 3 Key Laboratory of Environmental Remediation and Ecological Health, Ministry of Education, Zhejiang University, Hangzhou, China; 4 Guangxi Zhuang Autonomous Region Institute of Meteorological and Disaster-Mitigation Research, Nanning, China; Clemson University, UNITED STATES

## Abstract

Most areas planted with sugarcane are located in southern China. However, remote sensing of sugarcane has been limited because useable remote sensing data are limited due to the cloudy climate of this region during the growing season and severe spectral mixing with other crops. In this study, we developed a methodology for automatically mapping sugarcane over large areas using time-series middle-resolution remote sensing data. For this purpose, two major techniques were used, the object-oriented method (OOM) and data mining (DM). In addition, time-series Chinese HJ-1 CCD images were obtained during the sugarcane growing period. Image objects were generated using a multi-resolution segmentation algorithm, and DM was implemented using the AdaBoost algorithm, which generated the prediction model. The prediction model was applied to the HJ-1 CCD time-series image objects, and then a map of the sugarcane planting area was produced. The classification accuracy was evaluated using independent field survey sampling points. The confusion matrix analysis showed that the overall classification accuracy reached 93.6% and that the Kappa coefficient was 0.85. Thus, the results showed that this method is feasible, efficient, and applicable for extrapolating the classification of other crops in large areas where the application of high-resolution remote sensing data is impractical due to financial considerations or because qualified images are limited.

## Introduction

Sugar is a major food additive and is one of the most important raw bioenergy materials. Sugar made from sugarcane accounts for approximately 80% of the total sugar production in China [1]. The areas of sugarcane planting and production in China are ranked third in the world after those of India and Brazil. Regarding safety and policy making, it is important to quickly estimate sugarcane planting status over large areas. Conventionally, the local government usually estimates the sugarcane planting area by using a field survey; however, the survey coverage is usually very limited and time consuming.

As a powerful alternative, remote sensing provides an effective method for monitoring crop growth and estimating crop yield due to its unique capabilities in terms of its spectral, temporal and spatial resolutions [2]. In the literature, many different types of optical remote sensing data, such as the Moderate Resolution Imaging Spectroradiometer (MODIS), Advanced Spaceborne Thermal Emission and Reflection Radiometer (ASTER), Landsat-5 Thematic Mapper (TM), High Resolution Imaging Camera (CCD) on board of China-Brazil Earth Resources Satellite-2 and -2B (CBERS-2 and -2B), Landsat-7 Enhanced Thematic Mapper Plus (ETM+), SPOT-5 High Resolution Geometrical (HRG), and ENVISAT Advanced SAR (ASAR), have been applied for discriminating between sugarcane varieties, mapping sugarcane planting areas and estimating sugarcane yields [1–6].

Nevertheless, approximately 90% of China’s sugarcane crop is grown in southern and southwest regions [1] where the landscape is highly heterogeneous and is covered by cloudy weather during the sugarcane growing season. Consequently, only a few qualified remote sensing images are available. Additionally, cross cultivation in the above-mentioned sugarcane growing regions is common; thus, the extraction of sugarcane information from remote sensing data is compromised by spectral mixing with other types of crops [7].

The unique phenology of sugarcane, which is longer than rice and peanut and shorter than evergreen plants, such as banana and eucalyptus, may provide valuable information for remote sensing classification in the study area. By properly using time-series remote sensing images, the phenology of sugarcane, which can be used to differentiate the sugarcane planting area from the other land cover types, may decrease the interference of similar spectra from the other vegetation in the spectrum and increase the classification accuracy [8].

Conventional remote sensing classification algorithms, e.g., the unsupervised/supervised classifiers, the Iterative Self-Organizing Data Analysis Technique (ISOData), the Maximum Likelihood (ML) classifier, the Neural Network (NN) and the Support Vector Machine (SVM), are applied directly to pixels and do not consider contextual information [9–11]. However, pixel-based classification procedures, particularly those only using single imagery, may cause problems in automatic pattern recognition due to phenological crop variability, different cropping systems and non-uniform measurement conditions [12]. Alternatively, object-oriented techniques based on multi-temporal remote sensing images have been widely applied for land cover classification[13].

Compared with traditional pixel-based remote sensing classification methods, object-oriented methods (OOMs) consider the analysis of an “object in space” instead of a “pixel in space” [14]. The objects in OOMs have geographical features such as shape and length; texture features such as the gray level co-occurrence matrix (GLCM); and topological entities such as adjacency [15]. All of the attributes of a specific object form a knowledge base for the sample objects and can be applied in the classification process using data mining (DM) techniques [16].

DM is a separate stage within a process known as knowledge discovery in database (KDD) [17]. In the KDD process, decision tree (DT) classification techniques have been used to classify remote sensing data and have several advantages over the maximum likelihood method and artificial neural network algorithms. DT has the ability to handle data measured on different scales, but lacks any assumptions concerning the frequency distributions of data in each of the classes and the ability to handle non-linear relationships between features and classes [9]. For high-dimensional data, DT has no obvious advantages compared with the ANN and MLC classifiers [18]. However, the boosting method in machine learning is more robust than traditional DT. The basic idea of boosting is to create a highly accurate prediction rule by combining many relatively weak and inaccurate rules [19]. The AdaBoost (Adaptive Boosting) algorithm was the first practical boosting algorithm and remains one of the most widely used and studied data mining algorithms [20].

The small sun-synchronous satellites for environmental and disaster monitoring and forecasting (HJ-1 A/B) of China were launched in 2008, with a spatial resolution of 30 m, four spectral bands ranging from 0.43–0.90°μm and a revisit cycle of four days (the revisit cycle of the constellation is 2 days) [11]. Considering the influences of climate and the affordability of high spatial resolution remote sensing data, this study aimed to demonstrate the feasibility of using OOMs and the AdaBoost algorithm based on HJ-1 A/B data to classify sugarcane growing areas in regions with limited data and complex land cover. Thus, this study aimed to classify sugarcane production at a large regional scale in southern China. The phenological information regarding the major crops in the study area was used to facilitate the selection of remote sensing data and the interpretation of the results. The accuracy of this classification was evaluated using an independent validation data set.

## Materials and Methods

### Ethics statement

No specific permissions were required for the field investigation in Suixi County, China. We confirm that the field investigation in Suixi County did not involve endangered or protected species.

### Study area

**Fig 1:**
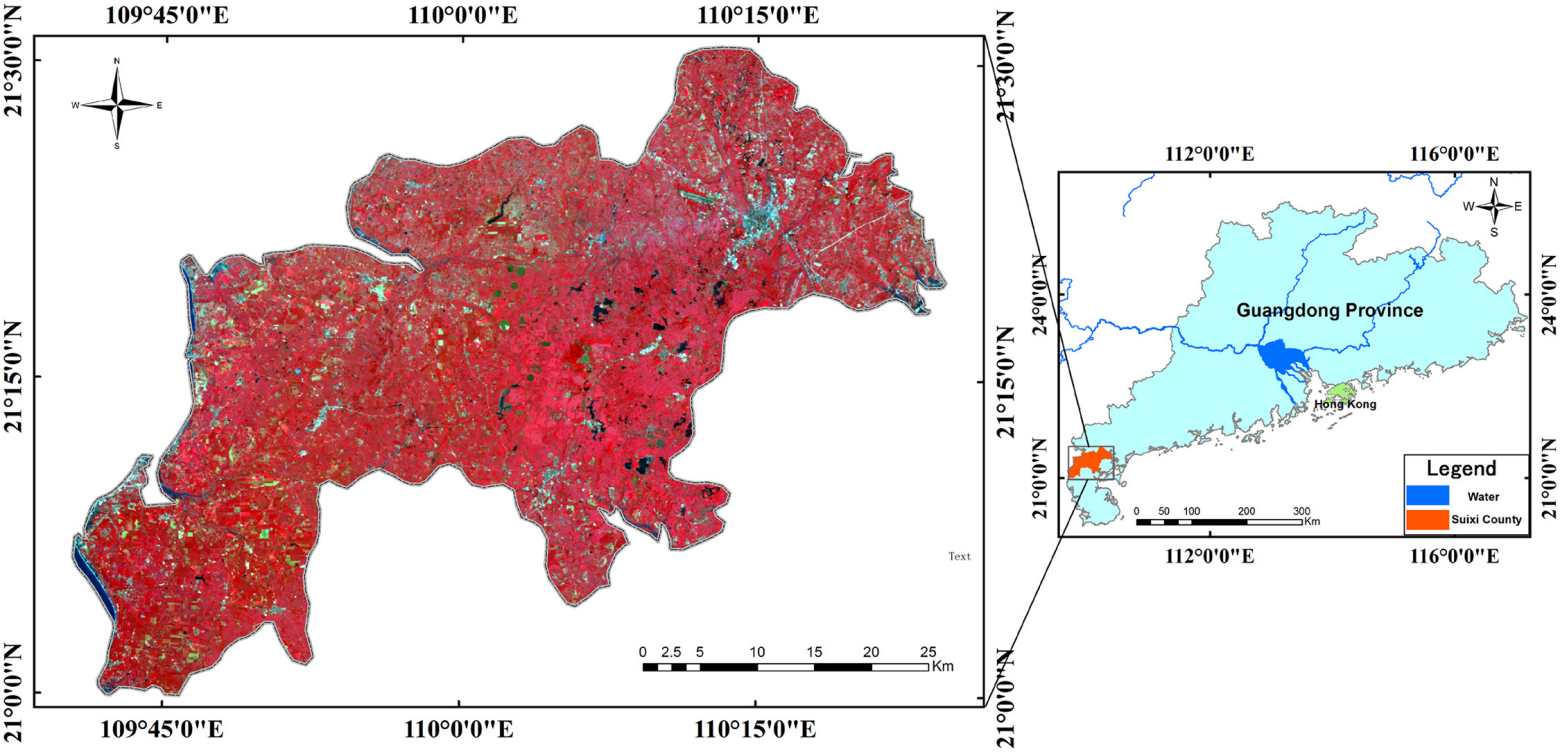
Study area in Suixi County, China. HJ-1 CCD image acquired on 26 October 2013 with a composition of R (4), G (3) and B (2). The HJ-1 CCD images were downloaded from the China Center for Resources Satellite Data and Application. I request permission for the open-access journal PLOS ONE to publish **Fig 1** under the Creative Commons Attribution License (CCAL) CC BY 3.0.

The study area, Suixi County, is located north of the Leizhou Peninsula in Guangdong Province (Fig 1). The terrain in this area is relatively flat, and the mean elevation is approximately 40 m above sea level. Suixi County has a subtropical maritime monsoon climate with a mean annual temperature of approximately 22.8°C and an annual precipitation between 1700–1800 mm. Suixi County is a major sugarcane planting area in Guangdong Province, with approximately 467 km^2^ of planted area year round. In addition to sugarcane, the major vegetation in this region includes rice, peanut, banana, grass, pineapple, pitaya, mango and eucalyptus. However, the dominant types of crops in terms of area are rice and peanut.

### Crop phenologies

Crop phenology is well correlated with changes in the spectral features of vegetation and is an important reference parameter for selecting remote sensing images [21, 22]. For sugarcane, the seedling period is from March to early June (including ratoon crops), the stem elongation period is from early June to the end of September, the sugar accumulation period is in October, the maturation period is from November to December, and harvest begins in late December and lasts until March of the next year. As mentioned above, of the other crops, only rice and peanut were considered because they cover large growing areas. The corresponding phenologies are listed in Table 1.

**Table 1:** Summary of the major phenological periods of the three major crops grown in the study area.

Crop	Year (2013)							Year (2014)				
	Jun.	Jul.	Aug.	Sep.	Oct.	Nov.	Dec.	Jan.	Feb.	Mar.	Apr.	May
Sugarcane	ST	ST	ST	ST	SA	MA	MA/HA	HA	HA	HA/SE	HA/SE	SL
Rice	HA	SO	VE	RE	MA	HA	FA	FA	FA	SO	VE	RE/MA
Peanut	MA/HA	SE	SL	FP	MA	HA	FA	FA	FA	SE	SL	FP

Note: HA: harvest stage; SE: seeding stage; SL: seedling stage; ST: stem elongation stage; SA: sugar accumulation stage; MA: maturation stage; SO: sowing-transplanting stage; VE: vegetative stage; RE: reproductive stage; FA: fallow; FP: flowering-podding stage.

### Remote sensing images

The sensor characteristics of the HJ-1 CCD images are presented in Table 2. To better differentiate sugarcane from the other land cover classes, 6 HJ-1 CCD images (Table 3) were obtained based on the sugarcane phenological periods shown in Table 1. The HJ-1 CCD images were geometrically corrected (the total root mean square error<0.5 pixel) by using Landsat-8 OLI data (acquired on 14/11/2014, path/row: 124/45) of the entire study area for the reference image. Atmospheric correction was performed using the FLAASH module in the ENVI package.

**Table 2:** Specifications of the HJ-1 A/B satellites.

Satellite	Payload	Band	Spectral range (μm)	Spatial resolution (m)	Swath width (km)	Revisit cycle (day)
HJ-1 A/B	Multispectral CCD camera	1	0.43–0.52	30	360 (700 for two)	4 (2 for constellation)
		2	0.52–0.60	30		
		3	0.63–0.69	30		
		4	0.76–0.90	30		

**Table 3:** HJ-1 A/B CCD images used in the classification.

No.	Satellite	Sensor	Date (dd/mm/yyyy)	Phenology of sugarcane
1	HJ-1 A	CCD2	13/06/2013	Stem elongation
2	HJ-1 A	CCD1	03/10/2013	Sugar accumulation
3	HJ-1 A	CCD1	26/10/2013	Sugar accumulation
4	HJ-1 B	CCD2	28/12/2013	Maturation
5	HJ-1 A	CCD1	23/01/2014	Harvest
6	HJ-1 B	CCD2	13/05/2014	Seedling

#### Classification methods

**Fig 2:**
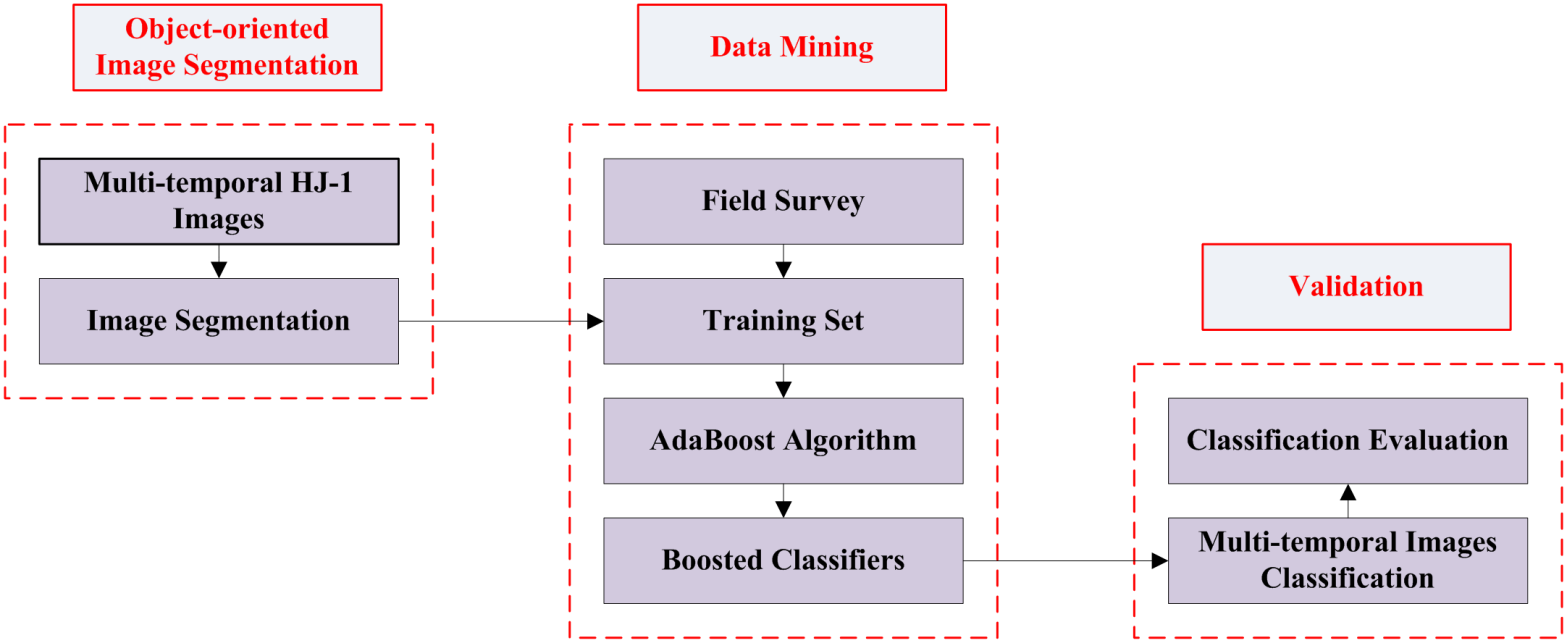
Schematic diagram illustrates the primary processes in the extraction of sugarcane growing areas. I request permission for the open-access journal PLOS ONE to publish **Fig 2** under the Creative Commons Attribution License (CCAL) CC BY 3.0.

The proposed approach includes two major steps: image segmentation and data mining. The first part includes object-oriented multi-resolution image segmentation and attribute table generation, and the second part includes building the training set, using the AdaBoost algorithm and boosted classifiers, and interpreting and evaluating the classification results (Fig 2).

#### Image segmentation

Object-oriented image segmentation was performed using the eCognition software [23]. The generation of objects was subjected to the heterogeneity criteria by adjusting the spectral band weight, scale parameter, form factor, and compactness factor. To guarantee the homogeneity of objects, all bands of the HJ-1 CCD time-series images were selected for segmentation. The object attributes used in the following classification are shown in Table 4. The selected attributes were spectral, spatial, textural and customized attributes, such as the normalized difference vegetation index (NDVI) [24], which has been demonstrated as closely correlated with the leaf area, biomass, percent ground cover and crop productivity [25, 26]; the enhanced vegetation index (EVI) [27]; and the 2-band enhanced vegetation index (EVI2) [28].

**Table 4:** Object attributes used to characterize sugarcane and the other classes in the selected HJ-1 CCD time-series images.

Type	Attribute	Description	Reference value range
Customized	NDVI	Vegetation index	[-1, 1]
	EVI	Vegetation index	[-1, 1]
	EVI2	Vegetation index	[-1, 1]
Spectral	Mean	Mean spectral intensity of an image object	$\left[C_{k}^{\text{min}},C_{k}^{\text{max}}\right]$ ^a^
	Standard deviation	Standard deviation of the spectral intensity of an image object	$\left[0,\frac{1}{2}C_{k}^{range}\right]$
Spatial	Area	Total area of an image object	[0, scene size]
	Border length	Sum of outer and inner borders, if existing, of an image object	[0, ∞]
	Pixel number	Number of pixels forming an image object	[0, scene size]
	Asymmetry	Relative length of an image object compared to a regular polygon	[0, 1]
	Border index	Ratio between the border length of the image object and the smallest enclosing rectangle	[1, ∞], 1 = ideal
	Compactness	Product of the length and width divided by the number of pixels of the image object	[0, ∞], 1 = ideal
	Elliptic fit	Describing how well an image object fits into an ellipse of similar size and proportion	[0, 1], 1 = complete fit, and 0 = no fit
	Main direction	Direction of the eigenvector belonging to the larger of the two eigenvalues derived from the covariance matrix of the spatial distribution of the image object	[0, 180]
	Rectangular fit	Describing how well an image object fits into a rectangle of similar size and proportion	[0, 1], where 1 represents a perfect rectangle
	Roundness	Describing how similar an image object fits into an ellipse	[0, ∞], 0 = ideal
	Shape index	Describing the smoothness of the surface of an image object border	[0, 1]
Texture	GLCM^b^contrast	Measure of the amount of local variation in the image	[0, 65025]
	GLCM homogeneity	Opposite of the contrast	[0, 1]
	GLCM dissimilarity	Similar to contrast but increases linearly	[0, 255]
	GLCM entropy	A measure of textural uniformity of an image	[0, 10404]
	GLCM mean	Average intensity of all pixels in a 2-D image object that belonged to the GLCM	[0, 255]
	GLCM std. dev	Standard deviation of all pixels in a 2-D image object that belonged to the GLCM	[0, 255]
	GLCM correlation	Linear dependency of gray levels of neighboring pixels	[0, 1]

^*a*^ $C_{k}^{\text{min}}$: the darkest possible intensity; $C_{k}^{\text{max}}$: the brightest possible intensity.

^*b*^GLCM: gray level co-occurrence matrix.

#### Training data sets

A field campaign was conducted in 2014 in the study area, and 382 field samples were collected using a portable GPS (Trimble SA). Among the samples, 146 were sugarcane, while the rest of the samples belonged to the other land cover types, e.g., water body, impervious surface/buildings, and other types of vegetation.

#### Data mining

Data mining involv.es the selection and application of intelligent techniques to extract patterns of interest for the effective production of knowledge [17]. In this study, the overall goals of data mining were to extract information from a data set (i.e., the 382 samples) and transform it into an understandable structure for further use. For this purpose, the boosting technique was applied.

Boosting is a machine learning ensemble meta-algorithm that can be used to reduce bias and variance in supervised learning. The basic principle of boosting is to learn multiple classifiers (weak classifiers) by changing the weights of the training samples and then combine these classifiers to improve classification performance. In this study, we used the AdaBoost algorithm.

AdaBoost is a generic iterative supervised learning algorithm that combines the other learning algorithms (weak learners) into a weighted linear boosted classifier to obtain a much higher accuracy [29]. Itis the first practical boosting algorithm and works by changing the weights of training data at each iteration (i.e., increasing the weights of the misclassified samples in the previous weak classifier and reducing the weights of the correctly classified samples). Thus, the misclassified samples will receive more attention due to their increased weights. Using this method, we should obtain a series of weak classifiers. Second, the algorithm adopts a weighted majority vote strategy in which the weights of the weak classifiers with small classification error rates are increased to improve their importance in the vote and vice versa [20, 30]. The package 'adabag' in the R environment was used for this purpose [31].

To evaluate the classification model generated by the AdaBoost algorithm, a standard statistics tool known as cross validation was used to provide an objective measure of quality for the generated model [32]. Specifically, a k-fold cross-validation method was adopted. The k-fold cross-validation involves partitioning a data set into k randomly complementary subsets. Of the k subsets, the decision tree built from the remaining k-1 subsets (called the training sets) will be validated by the retained single one. The cross validation process is then repeated k times (the folds), with each of the k subsets used exactly once as the validation data. Additional details on the cross-validation concept may be found in [33, 34]. We used a 10-fold cross validation to test the prediction model and summarized information regarding the classification error, such as the mean absolute error and relative absolute error.

#### Classification of multi-temporal images and evaluation

Using the verified predict model generated by the AdaBoost algorithm, the segmented HJ-1 CCD multi-temporal series data were classified into two classes of interest, sugarcane and others, by using the rules defined by the attributes and their respective thresholds, which were identified by AdaBoost.

Another 500 randomly selected sampling points (not involved in the training) were used to evaluate the classification accuracy. The confusion matrix assessment method was applied, and the global accuracy and Kappa coefficients were evaluated.

## Results

### Image segmentation

**Fig 3:**
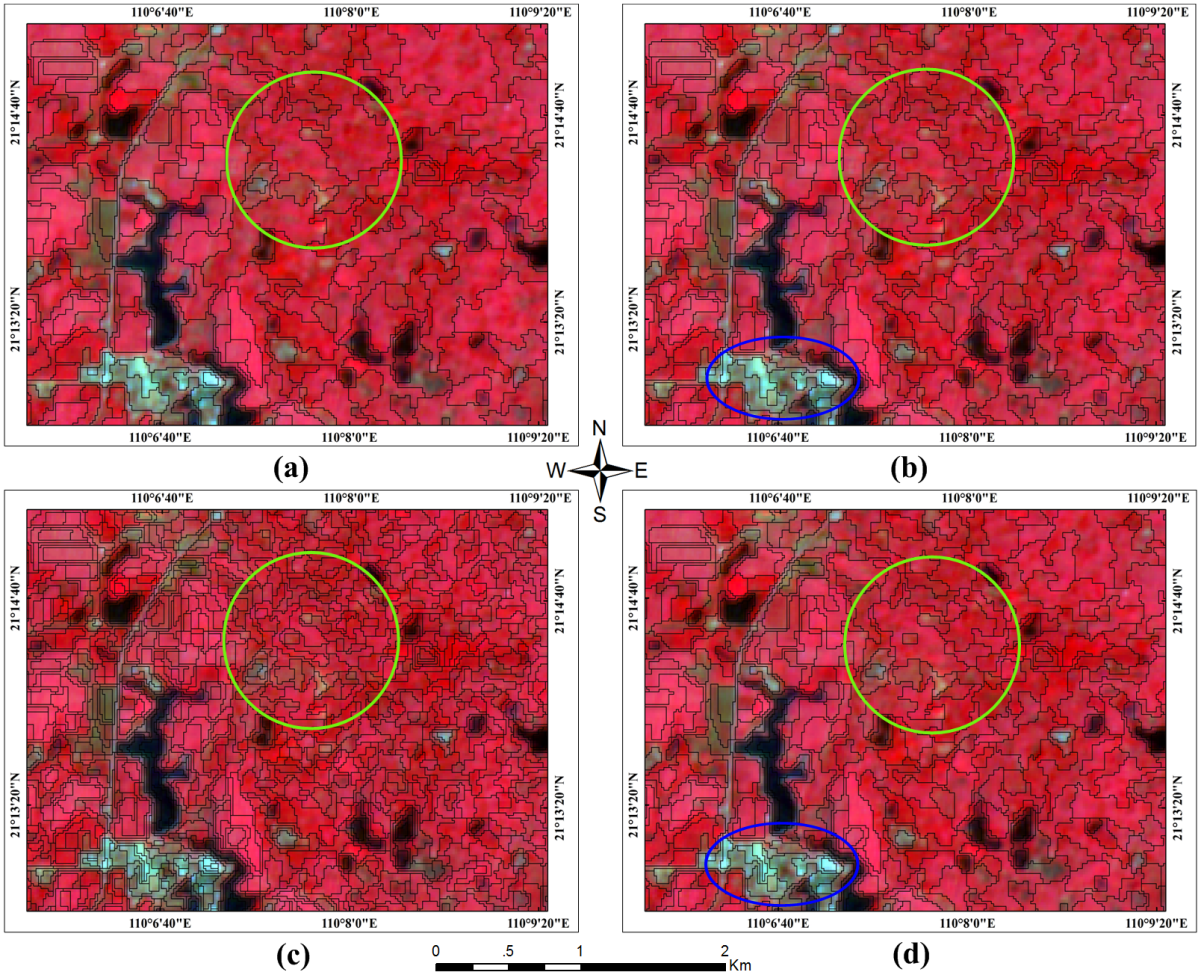
Multi-resolution segmentation using four different segmentation criteria. The base map of the multi-temporal HJ-1 CCD multi-spectral images with the following composition: R (4), G (3) and B (2). I request permission for the open-access journal PLOS ONE to publish **Fig 3** under the Creative Commons Attribution License (CCAL) CC BY 3.0.

In the process of segmenting the multi-temporal HJ-1 CCD time-series images, image objects were generated based on several adjustable criteria of homogeneity or heterogeneity in color and shape. The four parameters listed in Table 5 (i.e., scale, shape, color and compactness) need to be calibrated. We focused on adjusting the scale parameter because this parameter affects the average image object size (a larger value leads to larger objects and vice versa). To achieve better classification results, four different scale parameter values were used, and the results were compared using visual interpretation to determine the most suitable scale parameter value (Fig 3).

**Table 5:** Multi-resolution segmentation criteria used for the multi-temporal HJ-1 CCD images.

Parameter	(a)	(b)	(c)	(d)
Scale	50	40	30	35
Shape	0.2	0.2	0.2	0.2
Color	0.8	0.8	0.8	0.8
Compactness	0.5	0.5	0.5	0.5

We tuned the scale parameter using four different settings, i.e., 50, 40, 35 and 30. In Fig 3a, the segmentation was inadequate and the mixing of different types of cropland was severe (e.g., in the green circle). In Fig 3b, the pattern was more reasonable and the value of the scale parameter decreased to 40, which split the large mixed croplands into smaller mixed croplands. When the scale parameter decreased to 30, as shown in Fig 3c, over-segmentation occurred, indicating that further reducing the scale parameter would not improve the effect of segmentation. However, when the scale parameter was set to 35, as shown in Fig 3d, the segmentation effect showed no obvious changes (in green circles) compared with Fig 3b; however, the residential areas (in blue circles) were over-segmented. Thus, we selected the parameters in column (b) in Table 5 and segmented the HJ-1 CCD time-series images into 22,763 objects to form the test set (including the training set).

### Data mining

**Fig 4:**
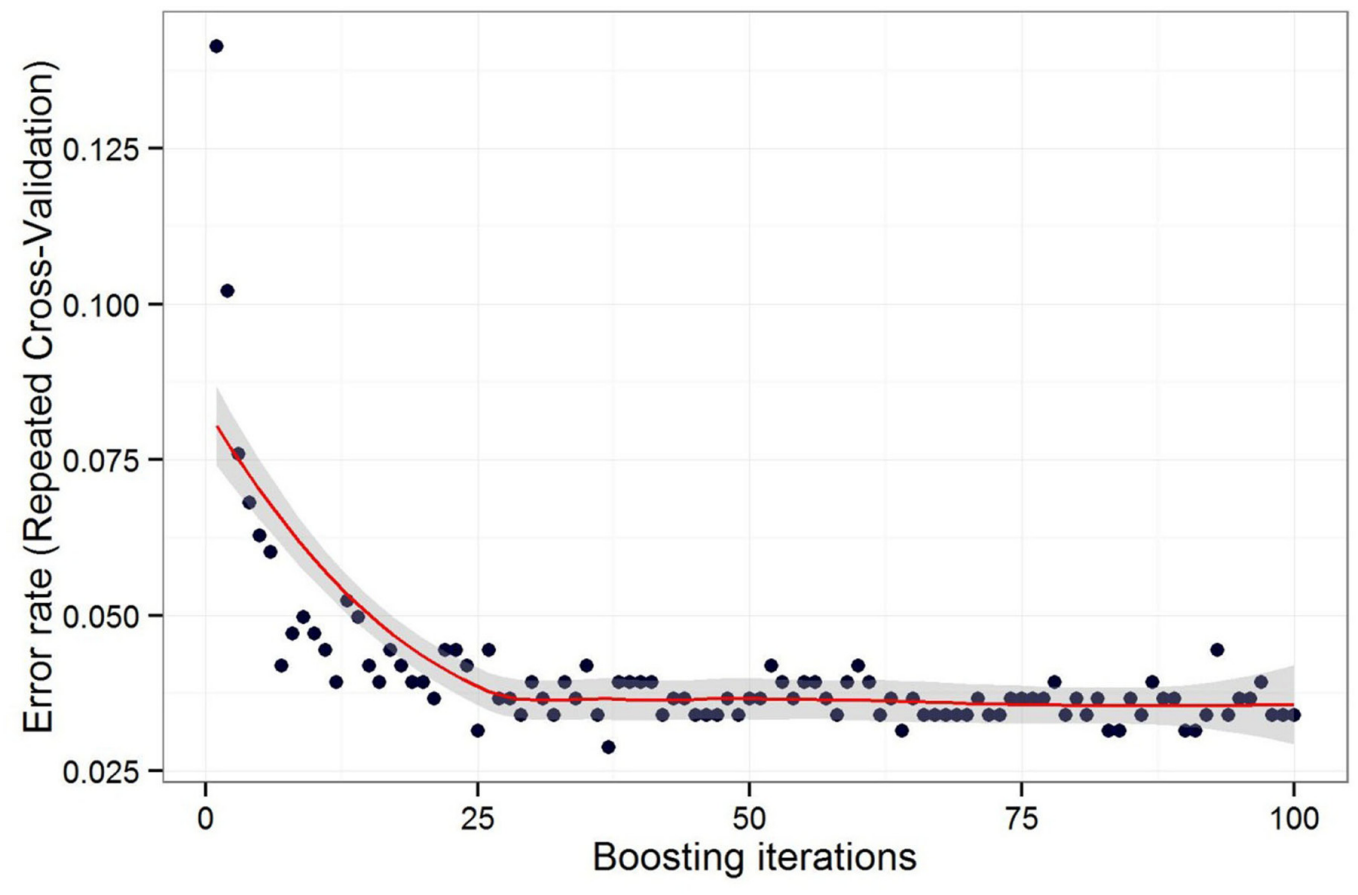
Relationship between the boosting iteration number and the corresponding error rate. I request permission for the open-access journal PLOS ONE to publish **Fig 4** under the Creative Commons Attribution License (CCAL) CC BY 3.0.

To determine suitable boosting iteration number ranges, we gradually increased the boosting iteration number from 1 to 100 and calculated the classification error rate as shown in Fig 4. The error rate decreased quickly as the boosting iteration number increased from 1 to 25. Beyond 25, increasing the boosting iteration number did not improve the error rate significantly, and the error rate was approximately 0.036.

**Fig 5:**
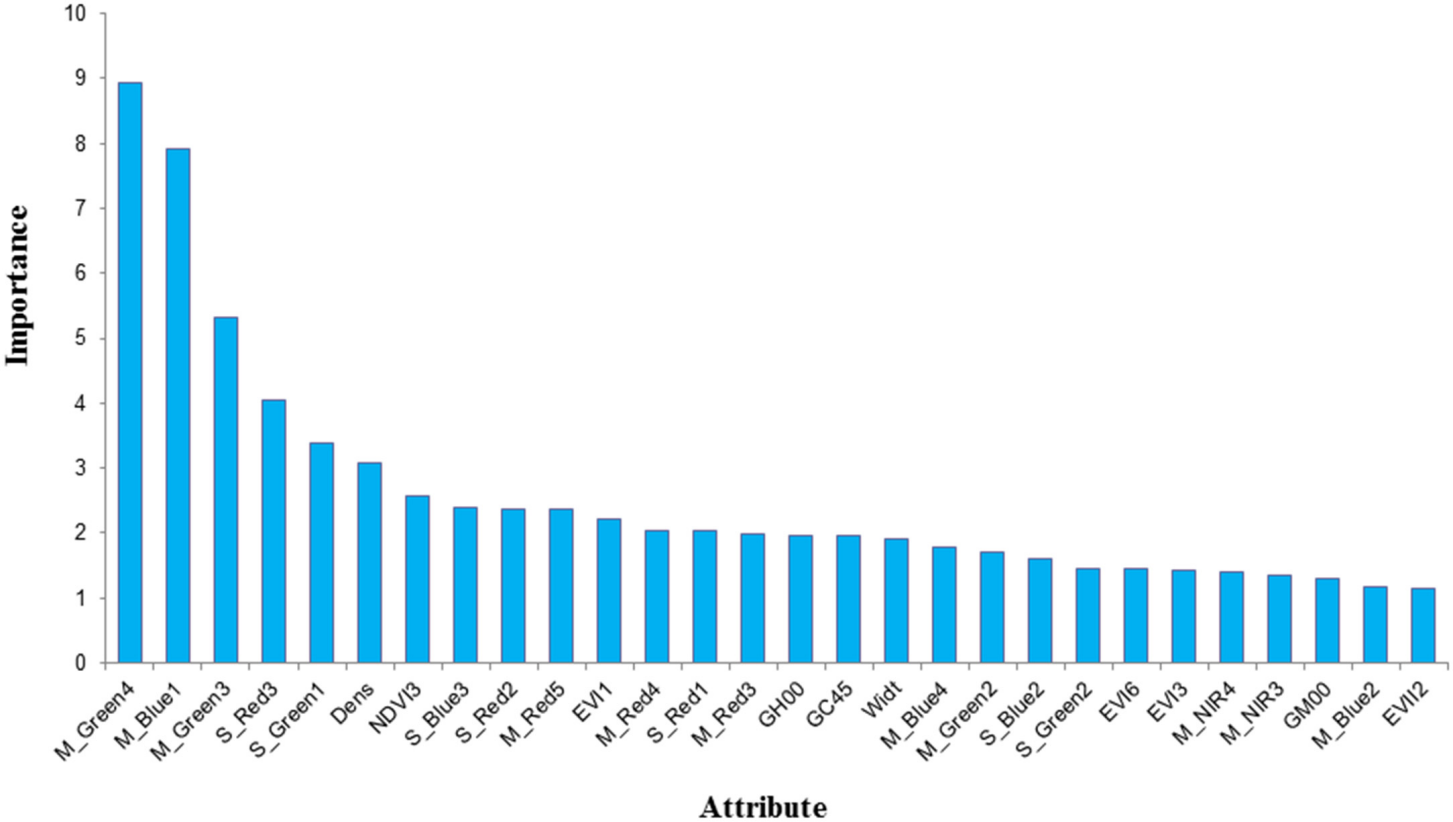
The relative importance of each attribute in the classification task (only those attributes with importance ≥ 1 are shown here). The nomenclature of the attributes are explained as follows. The prefix "M_" means the mean value of the specific spectral band that follows, e.g., Green, Blue, Red and NIR; the prefix "S_" represents the standard difference of the following band; and the suffix numbers, e.g., 1 to 6, correspond to the image numbers given in Table 3. The Dens, Widt and Asym are the abbreviations of Density, Width and Asymmetry, respectively. The GH00 is short for the GLCM homogeneity of an object at the angle of 0°; GC45 is short for the GLCM contrast of an object at the angle of 45°; and GM00 is short for the GLCM mean of an object at the angle of 0°. I request permission for the open-access journal PLOS ONE to publish **Fig 5** under the Creative Commons Attribution License (CCAL) CC BY 3.0.

We further tested the changes in the relative importance of each attribute in the boosting tree as the boosting iteration number increased from 100 to 1000. Fig 5 shows the relative importance of each attribute after 100 iterations. We increased the iteration number to 1000, retrieved the relative importance of each attribute again, and then compared our findings with the results shown in Fig 5. The ranks of the first four attributes remained unchanged, while the ranks of the other attributes (importance≥1) only exhibited minor changes. Thus, we ran the AdaBoost algorithm using 100 iterations, and the overall accuracy was 96.35% with a Kappa coefficient of 0.92.

### Decision rules

**Fig 6:**
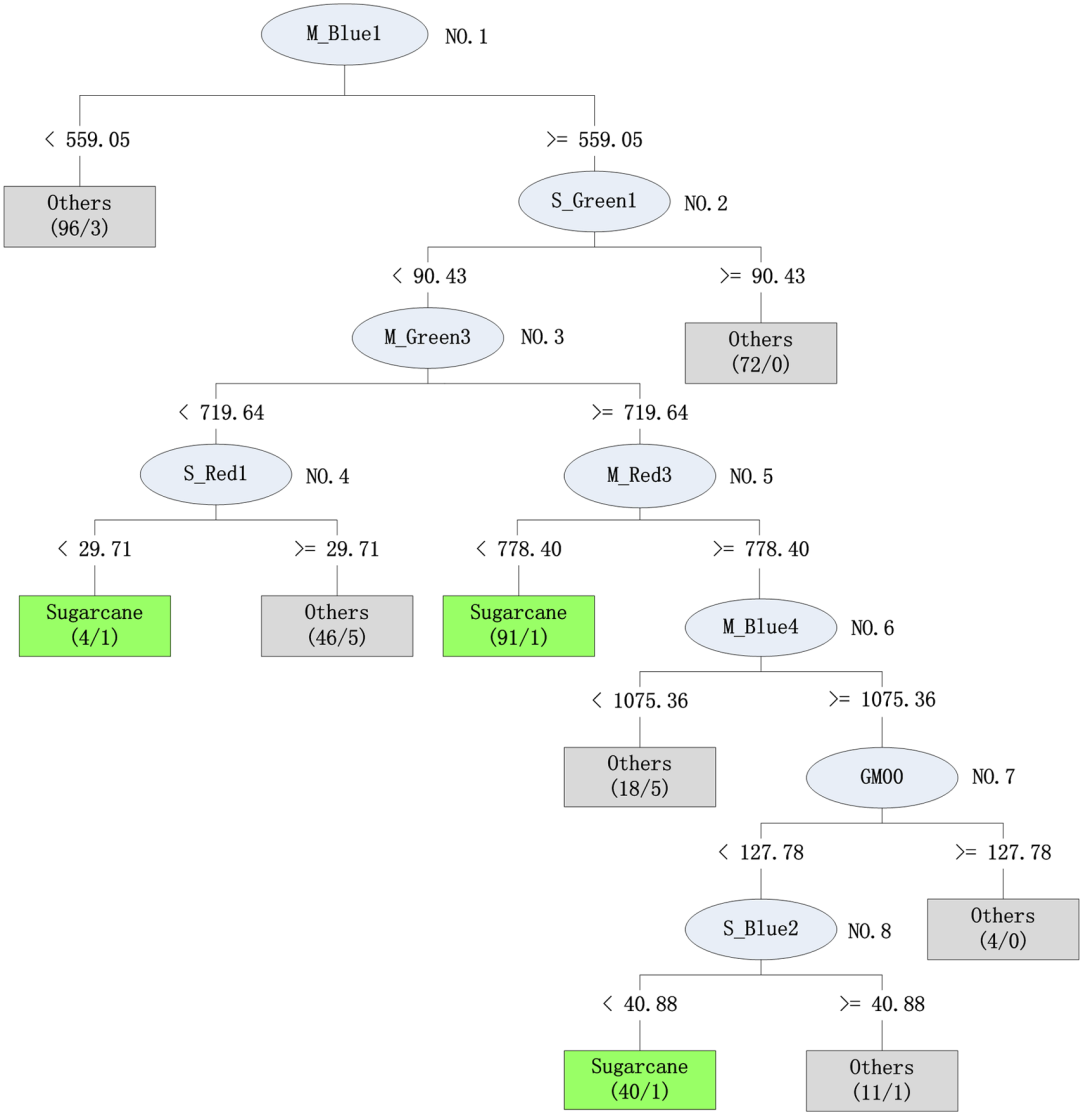
The decision tree with the largest weight generated by the AdaBoost algorithm to classify sugarcane and the other land cover types (note that the spectral reflectance was multiplied by a scale factor of 10^4^). I request permission for the open-access journal PLOS ONE to publish **Fig 6** under the Creative Commons Attribution License (CCAL) CC BY 3.0.

By applying the AdaBoost algorithm iteratively, 100 DTs were generated. We chose the DT with the largest weight to illustrate the reasonability of the generated decision rules from the HJ-1 multi-temporal CCD time-series images (Fig 6).

The root of DT was M_Blue1, the mean value (M) of the spectral reflectance in the blue band of the No. 1 HJ-1 data (see Table 3, and all the attributes will follow the same nomenclature) and most of the eucalyptus and banana samples fell on the left side of the DT. During this period, the sugarcane was in the early stem elongation stage with small plants and low chlorophyll content in the canopy; thus, the reflectance in the blue band was obviously higher than that of eucalyptus and banana.

The No. 2 node was S_Green1, which is the standard deviation (S) of the spectral reflectance in the green band of the No. 1 HJ-1 data, and most of the residential objects fell on the right side of the branch. For crop objects, the spectral reflectance in the green band and its standard deviation were much more uniform than those of the residential land (particularly the village, where scattered tree canopies usually mixed with houses).

The No. 3 node classified most of the sugarcane into the right branch. Except for several sugarcane objects, most of the samples (i.e., eucalyptus, banana and the mixture of evergreen forest and bare land) were classified into the right branch of the No. 4 node. Thus, at this stage (sugar accumulation for sugarcane, and maturation for rice and peanut), the average green band spectral reflectance was suitable for separating the major crops from the other plants.

At the same stage as the No. 3 node, the No. 5 node used the average red band spectral reflectance and classified a large portion of the sugarcane into the left branch. As mentioned above, the sugarcane was at its most vigorous growing stage (i.e., the sugar accumulation stage), and the relatively high chlorophyll content of the sugarcane canopy compared with the rice and peanut canopies decreased the spectral reflectance of the red band (the red band is a major chlorophyll absorption spectral region).

In December, i.e., the No. 6 node (HJ-1 image No. 4), the rice and peanut crops had been harvested and the corresponding croplands were in the fallow state. During this period, sugarcane harvesting had recently began, and 5 sugarcane samples were misclassified. However, most of the sugarcane samples, which had relatively higher blue band spectral reflectance, fell on the right branch.

Finally, the remaining sugarcane samples were identified by lower mean GLCM values and lower blue reflectance standard deviations in the No. 2 HJ-1 image, and bare lands and roads were classified as other objects with higher GLCM mean values. Additionally, the objects with higher blue reflectance standard deviations in the No. 2 HJ-1 image were classified as residential areas.

### Classification results

**Fig 7:**
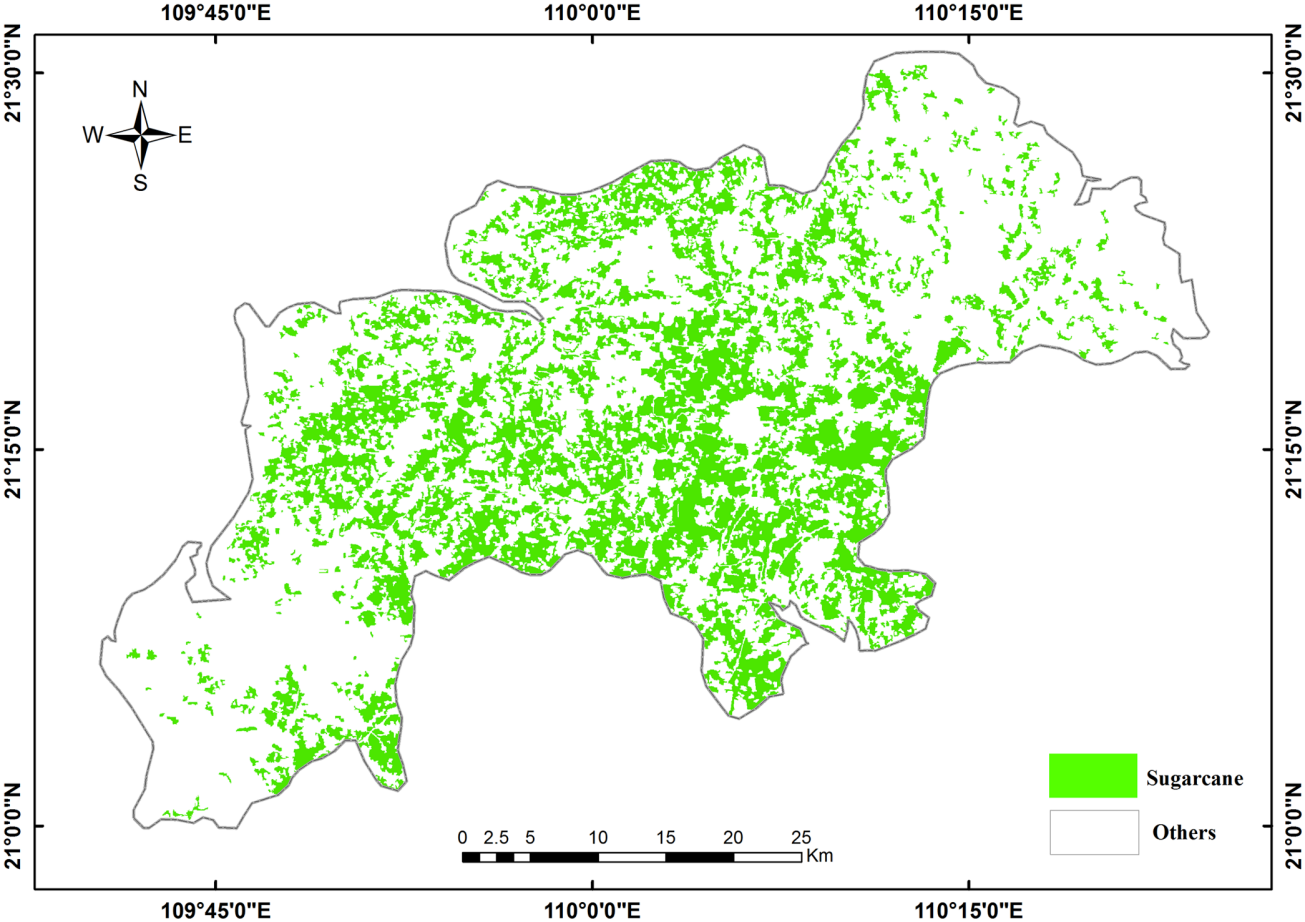
Thematic map of sugarcane areas in Suixi County. I request permission for the open-access journal PLOS ONE to publish **Fig 7** under the Creative Commons Attribution License (CCAL) CC BY 3.0.

The prediction model built by the AdaBoost algorithm was applied to the entire study area; the classification results for the sugarcane growing area are shown in Fig 7. The total growing area of sugarcane was approximately 481.58 km^2^ in the 2013–2014 harvest year. According to the statistical data of the local agriculture department in 2014, the total acreage of sugarcane was 492.97 km^2^, and the relative classification accuracy was approximately 97.68%.

The confusion matrix shown in Table 6 was generated using another 500 sampling points, which are mentioned in section 2.4.4. Among the 500 reference sampling points, 159 points belonged to the sugarcane class and 24 points were misclassified into the “others” class. Overall, 341 points belonged to the “others” class, and only 8 points were misclassified into the sugarcane class. Finally, the overall classification accuracy and Kappa coefficient were 93.60% and 0.85, respectively.

**Table 6:** Confusion matrix of the classification verified using independent sampling points.

	Reference map			
	Class	Sugarcane	Others	Total
Classification result	Sugarcane	135	8	143
	Others	24	333	357
	Total	159	341	500

## Discussions

### Image segmentation

Image segmentation is a fundamental step in object-oriented classification; however, the effectiveness of segmentation, which is determined by the segmentation parameter settings, heavily relies on the experience of the expert and the specific objects of interest [35]. In this study, we adopted a trial and error strategy, calibrated the scale parameter values from 50 to 30, and compared the different segmentation results. The results showed that setting the scale parameter to 50 is too loose and that setting the scale parameter to 30 is too tight. Furthermore, no obvious differences were observed when the scale parameter was set to 40 or 35 for croplands; however, when 35 was adopted, the fragmentation of residential land became severe and classification could not be performed. Thus, setting the scale parameter to 40 is appropriate for this specific problem.

### Data mining

The classification error rate decreased significantly and converged quickly when we used the AdaBoost algorithm (Fig 4). Compared with traditional DT classification, the ensemble classifier AdaBoost can effectively improve the classification accuracy.

The object attributes involved in the classification were generally classified as customized, spectral, spatial and texture (Table 4). Fig 5 only shows the 28 attributes with relative importance greater than 1 in the prediction model. Most of these attributes (18/28) belonged to the spectral category, followed by the customized (5/28), texture (3/28) and spatial (2/28) attribute categories. We speculated that the highly fragmented landscape in the study area (common in southern China) resulted in the extremely diversified geometric patterns, even for the same crops. The effects of the texture attributes on the classification were not significant and were potentially caused by the relative coarse spatial resolution of the HJ-1 CCD image data.

Among the 18 spectral attributes, 6, 5 and 5 attributes were related to the red, green and blue bands, respectively. Only two attributes (i.e., M_NIR4 and M_NIR3) were related to the NIR band, and the relative importance of these attributes was minor. This finding may be directly related to the low importance of vegetation indices (customized attributes) in the prediction model. Furthermore, this finding indicated that the combined single bands could achieve sound classification results without fully using the vegetation index information.

Regarding temporal attributes, the 4 (M_Blue1, S_Green1, EVI1 and S_Red1), 6 (S_Red2, M_Green2, S_Blue2, S_Green2, M_Blue2 and EVII2), 7 (M_Green3, S_Red3, NDVI3, S_Blue3, M_Red3, EVI3 and M_NIR3), 4 (M_Green4, M_Red4, M_Blue4 and M_NIR4), 1 (M_Red5) and 1 (EVI6) attributes were related to HJ-1 images 1–6, respectively, as shown in Table 3. These findings clearly show that the images from the early and middle phenology stages of sugarcane were more critical than the latter ones.

### Function of crop phenology in the classification

The phenologies of the major crops in the study area could provide key information for selecting remote sensing images [36]. The six images used in this study (Table 3) covered the entire sugarcane growing season and included the major phenologies of rice and peanut (Table 1). For example, the 1^st^ temporal period was June, when the blue band spectral reflectance could be used to clearly discriminate the crops (sugarcane, rice and peanut) from eucalyptus and banana. In Fig 6, the No. 5 node, which corresponds to the image from October, used the maximum red light absorption capability of sugarcane at that stage to differentiate between rice and peanut. When using knowledge of the phenologies of the major crops in the specific study area, the image selection should be very specific and the prediction model should be easy to interpret.

From Figs 5 and 6, we observed that the first four images dominated the building processes of the decision rules (i.e., the left two images might be redundant). Thus, under this technical framework, we built knowledge regarding which critical temporal window should be used to guide image selection. Next, the redundant images of these ranges could be safely omitted. For this specific study, if only several qualified images covering the early and middle sugarcane phenologies can be obtained, then the classification accuracy should be guaranteed. Additionally, the remote sensing images obtained during the latter phenologies after sugar accumulation may not be necessary.

## Conclusion

The classification of sugarcane in southern China in large areas faces two challenges: (1) a limited amount of qualified (and affordability in practical applications) remote sensing data due to pervasive cloudy weather and (2) the complex mixture of land cover and their similar spectral reflectances. In this context, our goal was to fully use the spectral and textural differences in various croplands in limited middle-resolution remote sensing images to facilitate the classification of sugarcane. Additionally, we aimed to determine whether a suitable temporal window exists to guide the selection of key remote sensing images in sugarcane classification.

In this study, six HJ-1 CCD images with a spatial resolution of 30 m and covering the entire sugarcane growing season (2013–2014) were used. The composite image, including the vegetation indices, was segmented into 22,763 objects using an object-oriented method. Next, the AdaBoost algorithm was used to build the DT model using 100 iterations. A 10-fold cross-validation method was applied to 382 field samples and showed that the overall classification and Kappa coefficient were 96.35% and 0.92, respectively. The DT model was applied to the entire study area to classify sugarcane and was tested using another 500 independent sampling points in the field. The overall accuracy achieved was 93.6%, and the Kappa coefficient was 0.85.

The classification model (i.e., a boosting tree) was built using the AdaBoost algorithm, and the categories and temporal features of the object attributes (Table 4) with relative importance greater than 1 were specifically checked (Fig 5). According to the proportion, most of the attributes belonged to the spectral category, followed by the customized, texture and spatial categories. Most of the spectral attributes were related to visible bands, and the effects of NIR bands and vegetation indices were minor. Only three texture and two spatial attributes with relative importance greater than 1 were identified, which might be caused by the relative coarse spatial resolution of the HJ-1 CCD data and the highly fragmented and irregular landscape in the study area.

Interestingly, we found that most of the attributes belonged to the early and middle temporal HJ-1 images and that the images during the early and middle phenology stages of sugarcane were clearly more critical compared with the latter images. Thus, under this technical framework, constructing an optimized image selection principle to guide remote sensing classification in similar regions is possible.
